# Multiyear evaluation of agronomic traits, nutritional quality, macro and microelement profiles of white maize genotypes (*Zea mays* L.) under Black Sea conditions

**DOI:** 10.3389/fpls.2026.1793293

**Published:** 2026-03-18

**Authors:** Erkan Özata, Barış Alaca, Gözde Hafize Yıldırım, Nora M. Al Aboud, Muhammad Tanveer Altaf

**Affiliations:** 1 Black Sea Agricultural Research Institute, Field Crops Department, Samsun, Türkiye; 2 Department of Field Crops, Faculty of Agriculture, Recep Tayyip Erdoğan University, Rize, Türkiye; 3 Department of Biology, College of Sciences, Umm Al-Qura University, Makkah, Saudi Arabia

**Keywords:** genotype x year interaction, GGE biplot, AMMI, grain yield, mineral elements, stability analysis, white maize

## Abstract

White maize (*Zea mays* L.) is increasingly valued for diversified food uses, yet agronomic performance and nutritional quality can fluctuate markedly across humid temperate seasons. This study evaluated 14 white maize testcross genotypes, including the commercial check P2948W, across five consecutive field seasons (2020-2024) in the Black Sea Region of Türkiye. Phenology and plant architecture, grain yield (t ha^-1^), major compositional traits (protein, oil, starch, cellulose and ash), and macro- and microelement concentrations (Ca, Mg, K, P, Fe, Zn, Cu and Mn) were assessed using near-infrared spectroscopy (NIRS) and standard field protocols. Data were analyzed using linear mixed-effects models to partition genotype, year, and genotype x year effects, followed by multivariate visualization (genotype x trait, GT, biplot) and stability assessment using AMMI and GGE biplot approaches (with years treated as environments). Grain yield showed wide genotypic variation (7.23-11.43 t ha-1), with P2948W ranking highest and TTBYM2019–37 lowest on the across-year mean basis, whereas pollen shedding occurred within a narrower window (73.7-78.1 days after planting). In contrast, most compositional traits and mineral means exhibited limited genotypic separation in the combined analysis, indicating strong seasonal influence on quality and mineral expression. Overall, the combined mixed-model and stability framework supports evidence-based selection of high-biomass, broadly adapted white maize candidates for regional cultivar development and provides a transparent basis for multi-year evaluation of quality and mineral attributes.

## 1 Introduction

White maize (*Zea mays* L.) is of substantial importance to human nutrition due to its high starch, protein, and dietary fiber content (Galal et al., 2025). This nutritional profile makes white maize a major staple food, particularly in developing countries, and enhances its value for both human consumption and animal feed (Nogalska et al., 2024). Maize is among the most widely cultivated field crops globally, with an annual production exceeding 1.1 billion tons and an area of approximately 194 million hectares (Yerli et al., 2024). This large-scale production clearly demonstrates maize’s central role in global food security and agricultural economics (Sanaev et al., 2024). Beyond human consumption, maize has a broad range of uses, including animal feed, bioenergy production, and industrial applications (Tang et al., 2025). The nutritional value of white maize varies with dry matter, crude protein, total sugars, ether extract, crude fiber, NDF, ADF, starch, and mineral composition, each of which determines its suitability for different utilization purposes (Nogalska et al., 2024). Such variability necessitates extensive research to establish optimal utilization strategies in both human diets and feed production (Mut et al., 2022; Serva, 2024). Consequently, evaluating the performance of diverse maize genotypes under varying environmental conditions, determining their adaptability, and optimizing nutrient composition provide critical information for breeding programs (Jan et al., 2025a; Popović et al., 2025). Agronomic traits such as grain yield and phenological development are key parameters that directly influence productivity and quality, offering essential insights into genotypic differences and environmental interactions (Sondarava et al., 2025; Jan et al., 2025b).

Based on endosperm color, maize is classified as white or yellow. Yellow maize is predominantly preferred for feed and industrial purposes (Serna-Saldivar, 2023), whereas white maize is primarily consumed as food and stands out as a staple, particularly in Africa and South America (Ranum et al., 2014). Because most global production targets feed industries, breeding programs have primarily focused on yellow maize varieties, resulting in lower yield in white maize genotypes. Although white maize is widely used for human nutrition, this preference is strongly shaped by cultural habits. For instance, in South Africa, yellow maize is sometimes perceived as a “poor man’s food,” prompting consumers to favor white maize (Aguk et al., 2021). Despite higher flour extraction rates in white maize than in yellow types, its fiber, vitamin, and mineral contents are generally lower (Ranum et al., 2014).

Because consumer preferences and processing need significantly influence maize demand, improving white maize should combine agronomic performance with end-use qualities and value chain requirements, rather than focusing solely on yield (Onu Ekpa et al., 2018; Pedro Revilla et al., 2022). In many areas, white maize is still mostly maintained as diverse open-pollinated populations, which tend to have lower yield potential and less uniformity compared to modern hybrids, underscoring the importance of targeted breeding that also preserves culturally favored product traits (Frank Kutka, 2011; Arushi Arora et al., 2024). Additionally, maize performance often depends on genotype x year interaction (years treated as environments), making multi-year and multi-location testing crucial for selecting locally adapted, high-yielding, and stable genotypes (Jan Bocianowski et al., 2024).

In Türkiye, white maize was first cultivated in the Black Sea Region in the 16th century and has since remained an essential component of regional agriculture and culinary traditions. Today, it is commonly used in bread, maize soup, and various local dishes (Kırtok, 1998). However, the white maize populations grown in the region are predominantly open-pollinated, resulting in lower yields than modern yellow hybrid varieties. As a result, current production levels do not adequately meet the region’s demand for white maize for traditional cuisine. Therefore, increasing the productivity of white maize has become both an economic and cultural necessity for the region.

This study aimed to evaluate the agronomic performance, fresh biomass and dry matter productivity, nutritional composition, and macro- and microelement profiles of selected white maize testcross genotypes grown under the ecological conditions of the Black Sea Region using multi-year data (2020-2024). Specifically, we sought to identify high-performing and stable genotype candidates suited to regional conditions and to provide genetic and selection insights to support future breeding efforts targeting high-biomass, quality-oriented white maize cultivars for food and feed uses.

## 2 Materials and methods

### 2.1 Plant material

In 2010, we collected more than 200 traditional white maize landraces from across Türkiye’s Black Sea Region and grouped this material into 20 populations. From 2010 to 2016, germplasm was advanced through repeated selfing to generate a set of inbred lines (ILs). After 2016, these ILs were test-crossed with two contrasting testers MO20 and P1610494 (a high-yielding USDA germplasm line) to quantify combining ability. Parental selection was then made chiefly on testcross grain yield, and the top 13 ILs were retained as elite parents. Experimental hybrids derived from these selected ILs were subsequently evaluated in multi-location trials conducted during 2020–2024 under Türkiye’s national cultivar registration system (**Supplementary Figure 1**; **Supplementary Table 1**).

### 2.2 Experiment site

The study was conducted over five consecutive growing seasons (2020, 2021, 2022, 2023, and 2024) in Çarşamba, Samsun, located in Türkiye’s central Black Sea region (41°13’ N, 36°39’ E; elevation: 2m). The area has a temperate climate with mild winters and warm summers, featuring minimal temperature variation daily and yearly. According to the Köppen-Geiger climate classification, the region is classified as ‘Cfa,’ characterized by mild winters, very hot summers and consistent precipitation throughout the year (Yerli et al., 2024). According to the International classification system, the field trial soil has a clay-loam (CL) texture, with 45,3% loam, 29,2% clay, and 25,5% sand. It has a slightly alkaline reaction and a medium calcareous structure. Available phosphorus is insufficient, and the organic matter content is low (IUSS working group wrb, 2015).

### 2.3 Experimental design and field management

The field trial followed the principles of a Randomized Complete Block Design (RCBD) with three replications. Maize was sown a total of 9 m^2^, with two rows on each plot, 70 cm between rows, and two rows in each plot length for a total of approximately 9 m^2^. Seeds were sown at 15 cm within-row spacing, resulting in 40 plants per row and 80 maize seeds per plot. Before sowing, 15:15:15 compound fertilizer was applied at a rate of 100 kg ha^-1^ of pure nitrogen (N), phosphorus (P_2_O_5_), and potassium (K_2_O). After sowing, 200 kg ha^-1^ urea fertilizer was applied. Field preparation involved autumn plowing followed by spring harrowing with a disc harrow. Sowing took place by hand on May 5–6 from 2020 to 2024. Drip irrigation was performed roughly every five days, with each event lasting until the soil reached field capacity. Irrigation was stopped at the dough stage.

Weed management was initiated through manual methods. Due to substantial weed pressure, hand weeding continued until maize plants reached the 3- to 5-leaf stage, at which point hoeing was implemented. Plots were harvested at the dough stage between 1 and 6 September in each season (2020-2024), depending on genotype. Grain yield (t ha^-^¹) was estimated from the net harvest area, excluding border rows and border plants. At harvest, three representative plants were removed for detailed sampling, and the remaining plants within the net plot were harvested for biomass determination. Total fresh weight from the harvested net area was recorded and converted to a hectare basis using the corresponding harvested area. For compositional and mineral assessments, a representative subsample of the chopped whole-plant material was collected immediately after harvest, thoroughly mixed to ensure homogeneity, and prepared for laboratory and NIRS analyses. Dry matter (DM) was determined from subsamples, and dry matter yield (t ha^-^¹) was calculated as grain yield (t ha^-^¹) × DM fraction.

### 2.4 Data collection of the genotypes’ yield and nutrient composition

Field-based phenotypic characterization was performed in accordance with the standard operating procedures issued by Türkiye’s Seed Certification Center (Tohumluk Tescil ve Sertifikasyon Merkez Müdürlüğü [TTSM], 2018). Nutrient composition analyses were performed using classical chemical methods. Total nitrogen (N) content of the plant samples was determined by the Kjeldahl wet digestion method and calculated using a nitrogen-to-protein conversion factor of 6.25. Crude oil content was measured by petroleum ether extraction using a Soxhlet-type apparatus, and cellulose was determined as crude fiber by sequential acid–alkali digestion followed by drying and ashing. Total starch content was determined by an enzymatic colorimetric method using thermostable α-amylase and amyloglucosidase to hydrolyze starch, followed by glucose quantification with a glucose oxidase–peroxidase reagent. Phosphorus (P), potassium (K), calcium (Ca), magnesium (Mg), iron (Fe), copper (Cu), zinc (Zn), and manganese (Mn) contents were determined after dry ashing. Dried and ground samples were ashed in a muffle furnace by gradually increasing the temperature to 500–550 °C until complete ashing was achieved (gray ash without carbonized particles). The ash was then dissolved in 4 mL of 3 N HNO_3_, and the resulting extracts were analyzed using ICP-OES (Inductively Coupled Plasma Optical Emission Spectrometry) (Huang and Schulte, 1985; Jones, 2001; Khan et al., 2022).

### 2.5 Statistical analysis

Statistical analysis. For each trait, linear mixed-effects models were fitted in R using lme4 (Bates et al., 2015) to quantify genotype (G), year (Y) and genotype x year (GxY) effects while accounting for the experimental design. For mean comparisons within the evaluated set of years, G, Y and GxY were tested as fixed effects, with replication nested within year modeled as a random effect. When estimating variance components and broad-sense heritability, a variance-component version of the same model was fitted with G and GxY treated as random effects. *Post-hoc* mean separation was performed using Tukey’s HSD at P < 0.05 where appropriate. Multi-trait relationships and stability were explored using genotype x trait (GT) biplot, AMMI and GGE biplot analyses, with years treated as the environments in the biplot frameworks.

Formula 1:


$$y=\;\mu +\;G_{i}+\;E_{j}+\;R_{r}+\;{\left(G\;x\;E\right)}_{ij}+\;\epsilon$$

Where:

- y: Phenotypic observation

- μ: Overall mean.

- $G_{i}$: Fixed effect of the i-th genotype.

- $E_{j}$: Fixed effect of the j-th environment (year: 2020, 2021, 2022, 2023, and 2024).

- $R_{r}$: Random effect of the r-th replication (nested within environment (year)).

- G x E: Fixed interaction effect between genotype and environment.

- ϵ: Residual error.

### 2.6 Heritability

Broad-sense heritability (H²) was estimated on an entry-mean basis across years using variance components from the random-effects model fitted to the combined dataset.

Variance components were derived from mean squares as follows:

$\sigma_{g}^{2}=(MS_{G}-MS_{G\times Y})/(n_{Y}\cdot r)$

$\sigma_{g\times y}^{2}=(MS_{G\times Y}-MS_{E})/r$,

$\sigma_{e}^{2}=MS_{E}$,

where, $MS_{G}$ is the genotype mean square, $MS_{G\times Y}$ is the genotype × year mean square, $MS_{E}$ is the residual mean square, $n_{Y}$ is the number of years, and r is the number of replications per year. Across-year H² was then computed as:

Formula 2:


$$H^{2}=\frac{\sigma_{g}^{2}}{\sigma_{g}^{2}+\sigma_{g\times y}^{2}/n_{Y}+\sigma_{e}^{2}/(n_{Y}\cdot r)}$$

Negative variance component estimates were constrained to zero.

## 3 Results

Broad-sense heritability (H²) estimates varied widely among traits and between the across-year and within-year analyses (**Table 1**). Across the five-year dataset (2020–2024), the highest and most consistent genetic signal was observed for Yield (t ha^-^¹) (H² = 0.623), indicating that genotype differences for this trait were relatively repeatable when averaged across years. Pollen DAP also showed a moderate across-year heritability (H² = 0.472). In contrast, Plant height and Kernel/Cob ratio exhibited low-to-moderate across-year heritability (H² = 0.336 and 0.345, respectively), while Ear height had a negligible across-year estimate (H² ≈ 0).

**Table 1:** This table reports broad-sense heritability (H²) estimates for each trait calculated (i) across the five-year dataset (2020–2024) and (ii) separately within each year (2020, 2021, 2022, 2023, 2024).

Trait	H2_across_years	2020	2021	2022	2023	2024
Pollen DAP	0.472	0.614	0.54	0.263	0.131	0.655
Plant Height (cm)	0.336	0.611	0.127	0.356	0	0.106
Ear Height (cm)	0	0.583	0	0.264	0	0.574
Kernel/Cob Ratio (%)	0.345	0	0.772	0.449	0.577	0.543
Yield (t ha-1)	0.623	0.907	0.907	0.151	0.964	0.053
Ash (%)	0	0	0.152	0	0.615	0
Fat (%)	0	0	0	0	0	0
Protein (%)	0	0	0	0.355	0.274	0.267
Cellulose (%)	0	0.305	0	0.428	0	0.043
Starch (%)	0	0.684	0.497	0.22	0.685	0
Fe (ppm)	0	0	0.267	0.132	0.096	0
Cu (ppm)	0	0.828	0.764	0.753	0.713	0.704
Mn (ppm)	0	0.263	0.307	0.447	0.075	0
Ca (ppm)	0	0	0.054	0	0	0
Mg (ppm)	0	0.54	0.277	0.345	0.153	0.347
K (ppm)	0	0.626	0.695	0.358	0.571	0.463
P (ppm)	0	0.589	0.553	0.283	0.678	0.066
Zn (ppm)	0.017	0.164	0.333	0.104	0.202	0.303

Estimates are based on 14 genotypes evaluated each year in a RCBD with two replications. The across-year H² values summarize the consistency of genetic differences among genotypes over years by accounting for genotype × year interaction and residual variation, while the within-year H² values reflect genetic repeatability within a single year under that year’s environmental conditions. Variance components were derived from the corresponding ANOVA/mixed-model partitioning of variation. When the genetic variance component was estimated as negligible (or negative due to sampling), it was constrained to zero; therefore, H² values shown as 0 indicate little detectable genetic variation for that trait in the given analysis.

Within-year estimates showed strong year-to-year shifts in genetic repeatability for several traits. Yield (t ha^-^¹) displayed very high H² in 2020 (0.907), 2021 (0.907), and 2023 (0.964), but much lower values in 2022 (0.151) and 2024 (0.053). Pollen DAP similarly ranged from low in 2023 (0.131) to high in 2024 (0.655). Kernel/Cob ratio was highly heritable in most years (e.g., 2021: 0.772; 2023: 0.577; 2024: 0.543) but essentially non-heritable in 2020 (0). Plant and ear height traits also fluctuated markedly by year, including near-zero estimates in some years. Quality and mineral traits generally showed very low across-year heritability, with several traits estimated at or near 0 across years (e.g., Ash, Fat, Protein, Cellulose, Starch, Fe, Cu, Mn, Ca, Mg, K, P), while Zn had a very small across-year estimate (H² = 0.017). Despite this, some mineral traits showed moderate-to-high within-year heritability in specific years—most notably Cu, which remained consistently high across all years (0.704–0.828), and K, P, and Mg, which were moderate in several years.

Different levels of variation were observed among the evaluated white maize genotypes for the investigated traits. Among phenological characteristics, Pollen DAP showed statistically significant differences among genotypes (p = 0.0115) (**Table 2**). The mean flowering time was 75.23 days, with a range of 73.7-78.1 days. In contrast, plant height and ear height did not exhibit significant genotype effects (p > 0.05). Grain yield, however, displayed highly significant genotypic variation (p = 0.0007**), with a cross-year mean yield values ranging from 7.23 to 11.43 t ha^-1^. The highest mean grain yield was recorded for P2948W, whereas TTBYM2019–37 consistently exhibited the lowest performance (**Table 2**).

**Table 2:** Mean values of Pollen DAP, plant height, ear height, yield, and quality components of maize genotypes grown between 2020 and 2024.

Genotype	Pollen DAP	Plant Height (PH, cm)	Ear Height (EH, cm)	Yield (t ha^-1^)	ASH (%)	OIL (%)	Protein (%)	Cellulose (%)	Starch (%)
P2948W	74.8	263.5	110	11.43	2.32	4.44	10.15	21.03	66.81
TTBYM2019-10	75.4	265.0	120	7.91	2.11	4.44	9.49	21.19	65.49
TTBYM2019-11	74.8	269.5	114	8.09	2.23	4.39	10.56	21.31	66.03
TTBYM2019-12	75.9	259.0	112	7.72	2.17	4.38	9.71	20.62	64.91
TTBYM2019-13	74.4	275.0	117.5	8.29	2.10	4.52	10.62	20.65	66.29
TTBYM2019-14	74.1	265.5	115.5	8.42	2.11	4.49	9.96	21.14	66.22
TTBYM2019-19	74.2	248.0	105	8.19	2.17	4.50	10.12	20.98	64.76
TTBYM2019-2	76.2	269.0	116.5	7.33	2.33	4.41	10.04	21.42	65.56
TTBYM2019-26	73.7	258.5	111.5	9.86	2.01	4.36	9.40	20.89	67.37
TTBYM2019-29	74.7	258.5	114.5	8.59	2.42	4.41	9.96	20.82	64.59
TTBYM2019-31	77.2	257.0	113	7.95	2.35	4.48	9.51	21.25	67.23
TTBYM2019-35	78.1	262.0	107.5	8.80	2.17	4.51	10.44	20.46	64.98
TTBYM2019-37	75.7	269.5	110.5	7.23	2.45	4.49	10.29	21.27	66.23
TTBYM2019-7	74	266.5	110.5	7.60	2.14	4.43	9.40	20.97	65.45
Mean	75.23	263.3	112.7	8.39	2.22	4.45	9.98	21.00	65.85
G	0.0115*	0.2366 ^ns^	0.4788 ^ns^	0.0007**	0.4859 ^ns^	0.9814 ^ns^	0.3398 ^ns^	0.5856 ^ns^	0.3872 ^ns^
Y	<.0001***	<.0001***	<.0001***	<.0001***	<.0001***	1.0000 ^ns^	0.5414 ^ns^	1.0000 ^ns^	1.0000 ^ns^
G x Y	0.1027 ^ns^	1.0000 ^ns^	1.0000 ^ns^	0.0006**	1.0000 ^ns^	1.0000 ^ns^	1.0000 ^ns^	1.0000 ^ns^	0.0832^ns^
CV (%)	7.45	8.52	13.76	23.46	28.44	6.29	13.38	4.85	4.00

Significance levels for the effects of genotype (G), year (Y), and genotype × year interaction (G × Y) were determined at the 5% significance level (P < 0.05). CV, coefficient of variation. Significance levels: ns, non-significant (P ≥ 0.05); *P < 0.05; **P < 0.01; ***P < 0.001.

**Figure 1:**
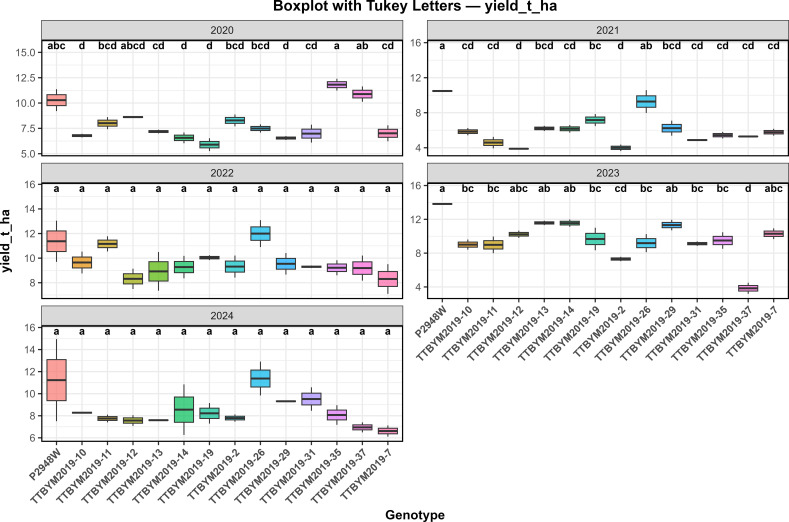
Year-wise boxplots of grain yield (yield_t_ha) across maize genotypes for the 2020–2024 growing seasons. Different lowercase letters above the boxplots indicate significant differences among genotypes within each year according to Tukey’s HSD test at the 5% significance level (P < 0.05).

Across the five-year evaluation period (2020-2024), clear differences in grain yield were observed among genotypes. Across-year mean yield ranged from 7.23 to 11.43 t ha^-1^ (**Table 2**). Year-wise comparisons indicated that genotype ranking shifted across seasons, reflecting a pronounced genotype x year (GxY) component (**Figure 1**). The commercial check P2948W frequently ranked among the upper-yield group across years, whereas TTBYM2019–37 tended to remain among the lower-yielding genotypes, highlighting the need for multi-year evaluation when selecting high-biomass candidates.

For compositional traits, no statistically significant differences were detected among genotypes for ash, oil, protein, cellulose, or starch contents (p > 0.05) (**Table 2**). The year factor exerted a strong influence on Pollen DAP, plant height, ear height, grain yield, and ash content (p < 0.0001***). In contrast, oil, protein, cellulose, and starch contents were not significantly affected by year. Genotype x year (GxY) interaction was significant only for grain yield (p = 0.0006**), while all other traits exhibited non-significant interactions (p > 0.05).

**Figure 2:**
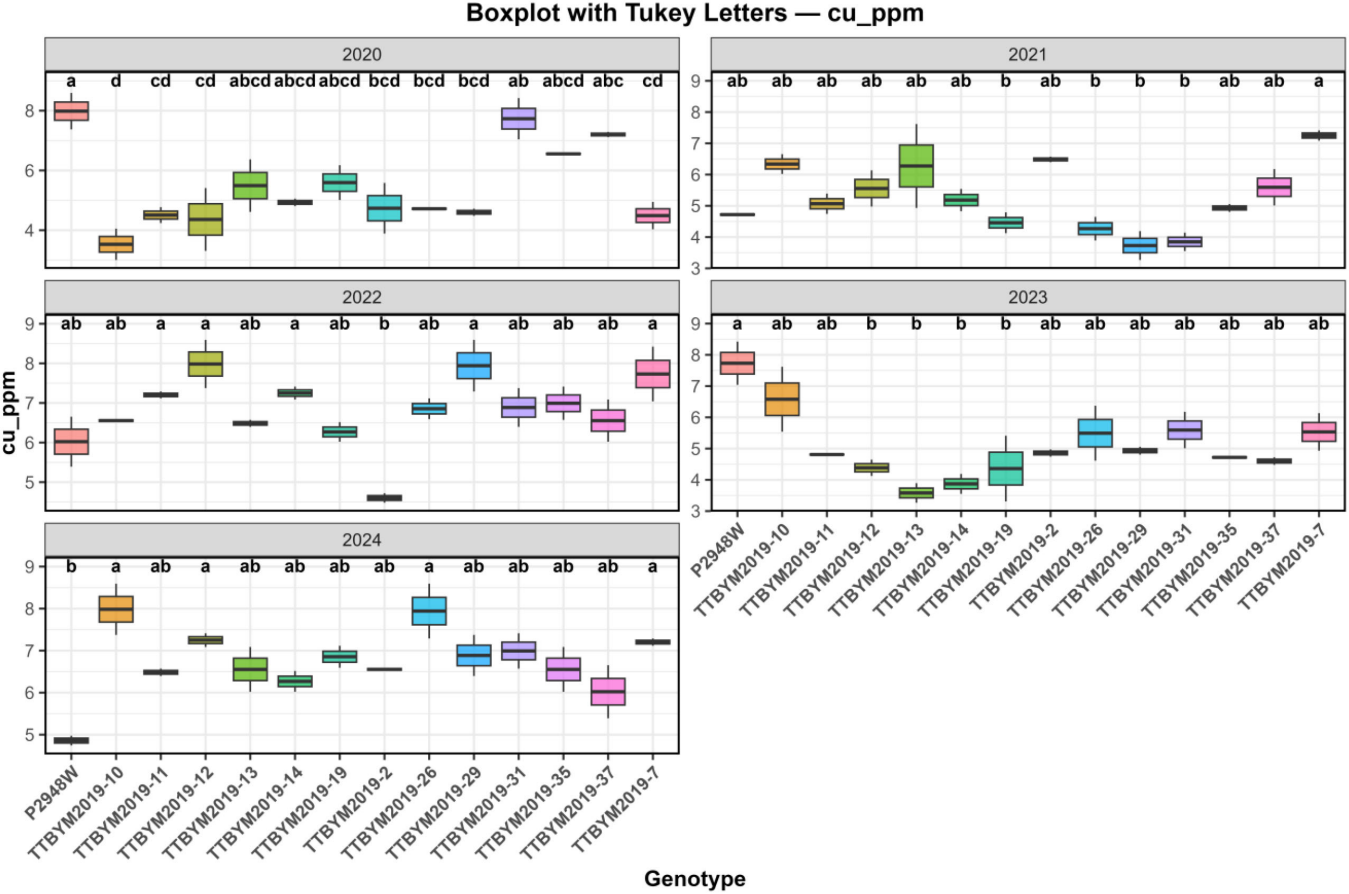
Year-wise boxplots of copper concentration (cu_ppm) across maize genotypes for the 2020–2024 growing seasons. Different lowercase letters above the boxplots indicate significant differences among genotypes within each year according to Tukey’s HSD test at the 5% significance level (P < 0.05).

**Figure 3:**
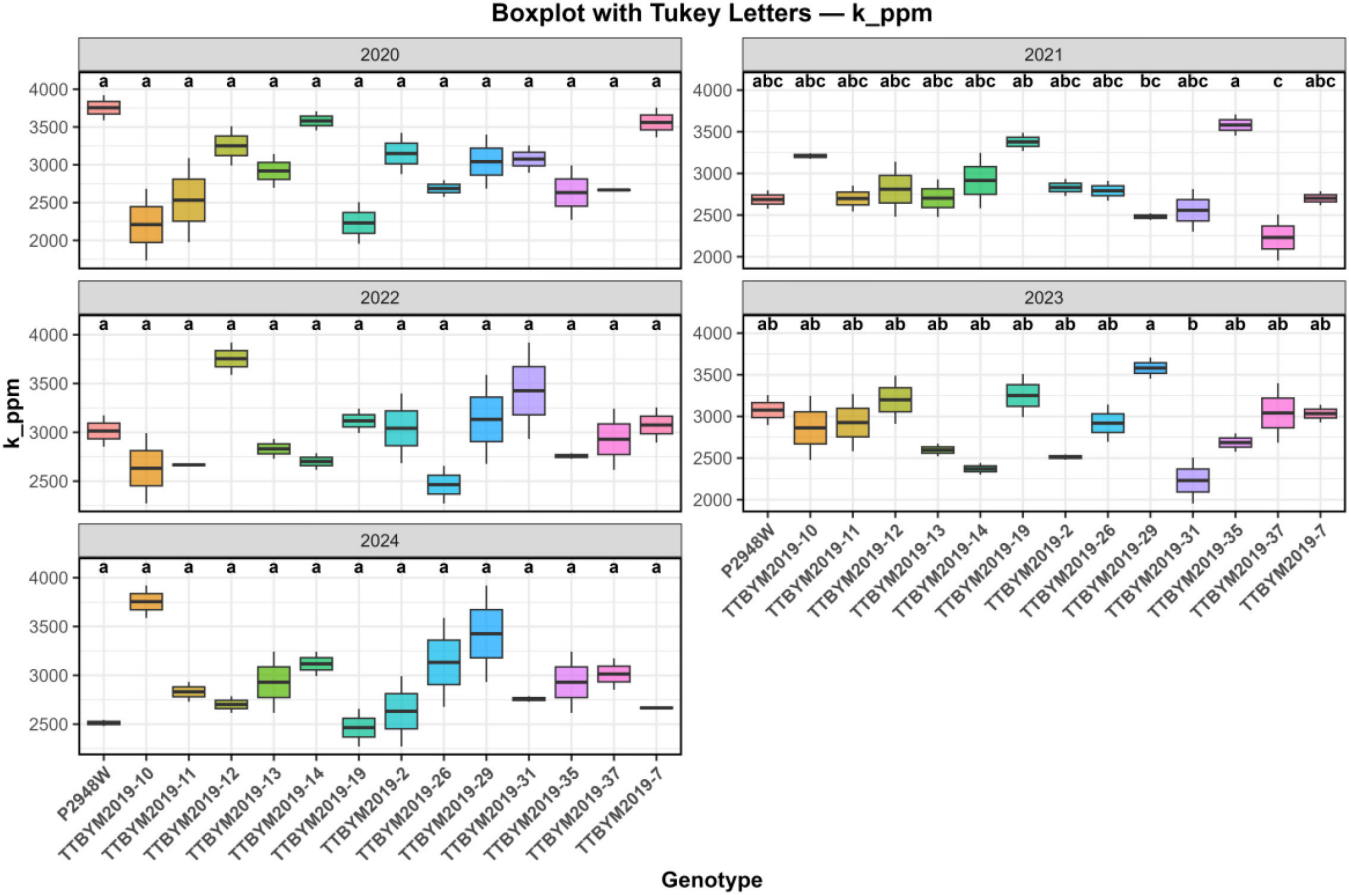
Year-wise boxplots of potassium concentration (k_ppm) across maize genotypes for the 2020–2024 seasons. Different lowercase letters above the boxplots indicate significant differences among genotypes within each year according to Tukey’s HSD test at the 5% significance level (P < 0.05).

Coefficients of variation differed considerably among traits. Ash (28.44%) and grain yield (23.46%) exhibited the highest variability, whereas starch (4.00%) and cellulose (4.85%) showed the lowest variation, indicating greater stability of major compositional traits across the evaluated seasons. Regarding macro- and microelement composition, numerical differences among genotypes were observed for all measured mineral traits; however, the combined analysis indicated no statistically significant genotype main effects for the overall mineral means (P > 0.05) (**Table 3**). In contrast, year and/or GxY effects were evident for specific elements, emphasizing that mineral expression is highly season-dependent and should be interpreted cautiously, with within-year comparisons used where supported by *post-hoc* tests (**Figures 2**, **3**). Copper content varied markedly across years. Over the five years, several genotypes (e.g., TTBYM2019-10, TTBYM2019–12 and TTBYM2019-14) tended to show higher numerical Cu concentrations in multiple seasons, whereas TTBYM2019–37 often showed lower values; nevertheless, these patterns were not expressed as significant genotype main effects in the combined analysis (**Figure 2**). Similarly, potassium content exhibited substantial seasonal variation. Some genotypes (e.g., TTBYM2019-12, TTBYM2019–13 and TTBYM2019-14) numerically maintained higher K levels in several years, while TTBYM2019–31 and TTBYM2019–37 were frequently lower, underscoring the dominant influence of year-to-year conditions on mineral profiles (**Figure 3**).

**Table 3:** Mean values of Fe, Cu, Mn, Ca, K, P, and Zn concentrations of maize genotypes grown between 2020 and 2024.

Genotype	Fe (ppm)	Cu (ppm)	Mn (ppm)	Ca (ppm)	Mg (ppm)	K (ppm)	P (ppm)	Zn (ppm)
P2948W	22.33	6.26	6.05	343.13	1071.87	3008.59	2674.48	24.50
TTBYM2019-10	20.74	6.20	5.48	154.80	1016.60	2933.39	2602.05	23.70
TTBYM2019-11	20.97	5.61	5.95	313.42	1050.58	2730.92	2505.76	24.83
TTBYM2019-12	20.63	5.91	4.91	471.68	1033.38	3143.38	2581.13	23.83
TTBYM2019-13	18.99	5.68	5.63	231.43	1062.33	2795.79	2586.47	24.15
TTBYM2019-14	21.46	5.50	5.59	209.56	1011.13	2937.29	2526.71	22.98
TTBYM2019-19	22.18	5.51	5.55	206.23	977.11	2888.63	2528.47	24.43
TTBYM2019-2	20.91	5.45	5.75	273.69	1079.71	2833.30	2661.44	26.44
TTBYM2019-26	22.66	5.86	5.25	251.27	1088.57	2798.89	2600.18	24.92
TTBYM2019-29	23.14	5.62	5.11	135.01	1107.80	3132.57	2807.54	23.89
TTBYM2019-31	20.93	6.21	6.26	176.98	1024.57	2809.63	2492.69	22.74
TTBYM2019-35	22.52	5.95	5.55	302.36	997.88	2917.50	2525.77	23.50
TTBYM2019-37	22.64	5.99	6.29	310.77	1090.94	2776.74	2674.74	24.79
TTBYM2019-7	23.39	6.44	5.60	541.74	1155.78	3007.73	2760.43	26.42
Mean	21.68	5.87	5.64	280.15	1054.88	2908.17	2609.13	24.37
G	0.7522 ^ns^	0.9085 ^ns^	0.3641 ^ns^	0.5188 ^ns^	0.4290 ^ns^	0.7863 ^ns^	0.7023 ^ns^	0.2530 ^ns^
Y	0.7594 ^ns^	<.0001***	1.0000 ^ns^	1.0000 ^ns^	1.0000 ^ns^	1.0000 ^ns^	0.6702 ^ns^	0.2376 ^ns^
G x Y	1.0000 ^ns^	<.0001***	1.0000 ^ns^	1.0000 ^ns^	0.1888 ^ns^	0.0037**	0.0096**	0.7271 ^ns^
CV (%)	21.89	19.89	22.58	140.78	14.03	15.48	11.98	13.30

Significance levels for the effects of genotype (G), year (Y), and genotype × year interaction (G × Y) were determined at the 5% significance level (P < 0.05). CV, coefficient of variation. Significance levels: ns, non-significant (P ≥ 0.05); *P < 0.05; **P < 0.01; ***P < 0.001.

**Figure 4:**
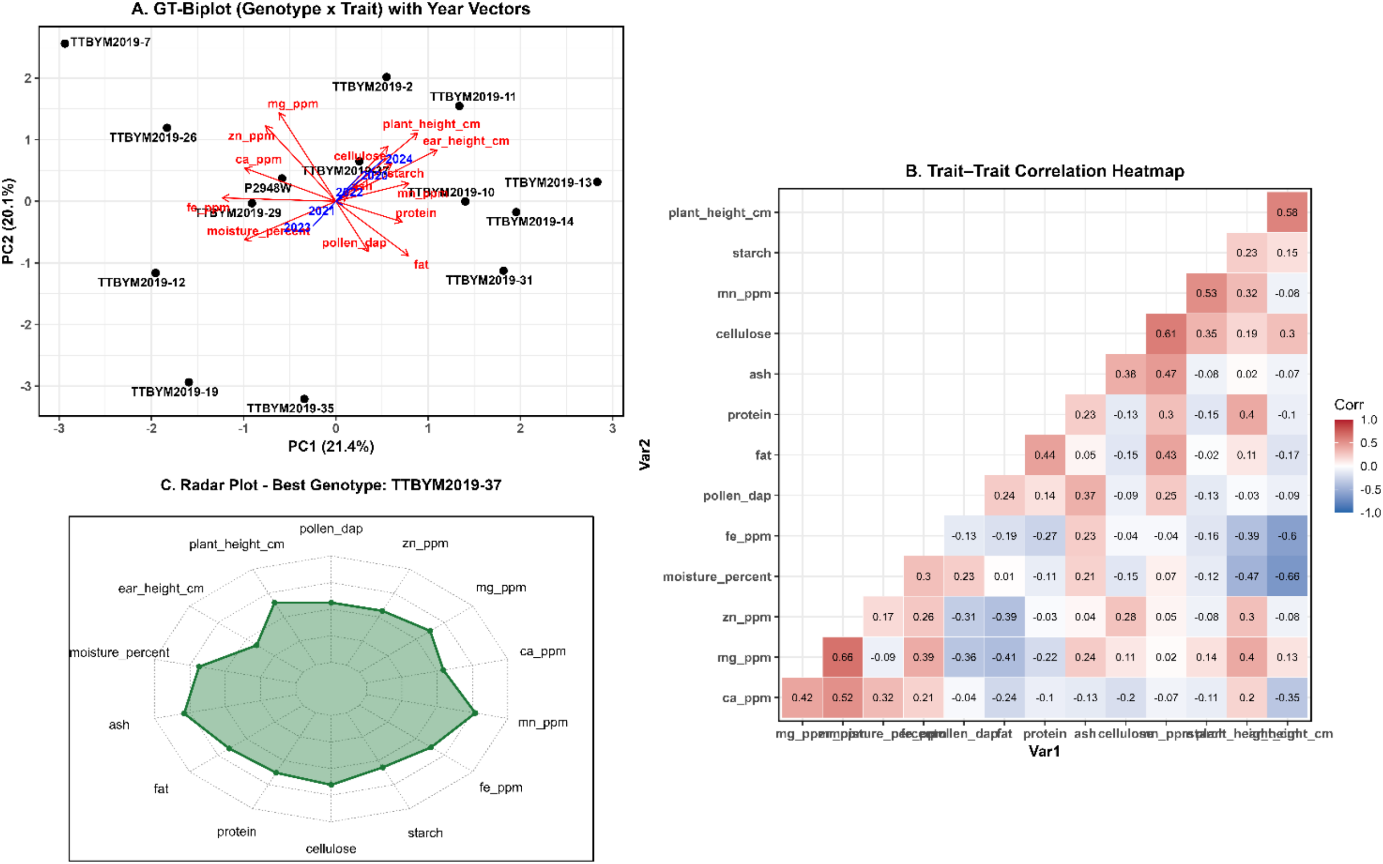
**(A)** GT-Biplot (Genotype x Trait) with year vectors showing the relationships among maize genotypes, traits, and yearly environments. **(B)** Trait-trait correlation heatmap illustrating pairwise Pearson correlation coefficients among phenological, agronomic and compositional traits. **(C)** Radar plot displaying the multi-trait performance profile of a representative genotype (TTBYM2019-37).

**Figure 5:**
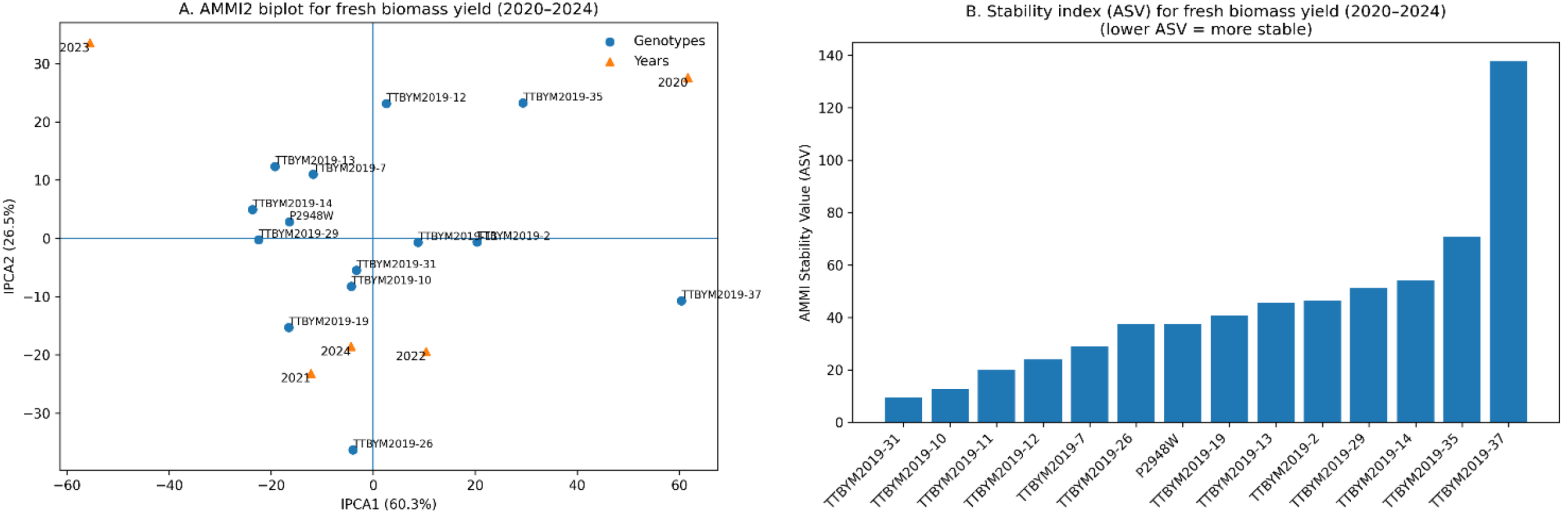
AMMI2 biplot and stability index for grain yield (t ha^-^¹) across five years (2020–2024). **(A)** AMMI2 biplot showing genotype and year scores on the first two interaction principal component axes (IPCA1 and IPCA2); percentages on axes indicate the proportion of the genotype × year (GE) interaction explained by each axis. **(B)** AMMI Stability Value (ASV) for each genotype (lower ASV = higher stability), summarizing yield stability across years based on IPCA1 and IPCA2 scores.

The GT-biplot explained a substantial proportion of the total variation, with PC1 = 21.4% and PC2 = 20.1% (**Figure 4A**), and revealed coherent trait groupings and directional associations among traits and years. The trait–trait correlation heatmap supported these patterns (**Figure 4B**), showing a positive association between plant height and ear height (r = 0.58) and strong positive relationships between cellulose and Mn (r = 0.61) as well as Mn and starch (r = 0.53). A pronounced positive association was also observed between Mg and moisture (r = 0.66). In contrast, ear height showed negative relationships with Fe (r = -0.60) and moisture (r = -0.66), indicating opposing trends between these traits in the evaluated dataset. The radar plot provides a compact summary of the standardized multi-trait profile of the selected “best genotype” (TTBYM2019-37) across agronomic, compositional, and mineral attributes (**Figure 4C**). The AMMI-based evaluation of grain yield across five years (2020–2024) is represented in **Figure 5**. The AMMI2 biplot indicated a strong genotype × year (G×Y) interaction, with the first two interaction axes explaining most of the GE variation (IPCA1 = 60.3% and IPCA2 = 26.5%) (**Figure 5A**). Years were clearly separated in the AMMI2 space, reflecting contrasting seasonal yield responses, and genotypes located farther from the origin showed stronger year-specific interaction effects. The AMMI Stability Value (ASV) provided a quantitative stability ranking (lower ASV = greater stability) (**Figure 5B**). The most stable genotypes were TTBYM2019–31 and TTBYM2019-10, followed by TTBYM2019–11 and TTBYM2019-12, whereas TTBYM2019-37 (and subsequently TTBYM2019-35) exhibited the highest ASV values, indicating the greatest yield instability across years.

## 4 Discussion

Across the five-year evaluation, yield showed the most repeatable genetic differences (H² = 0.623) and pollen DAP exhibited moderate repeatability (H² = 0.472), whereas several traits had low or near-zero across-year H² (**Table 1**), indicating stronger environmental influence and/or genotype × year (G×Y) effects that can obscure stable genetic variance when data are pooled (Holland et al., 2003). The large swings in within-year H² (e.g., yield high in 2020–2021 and 2023 but low in 2022 and 2024) reinforce that heritability is environment- and design-dependent, so single-year selection may be unreliable for environmentally sensitive traits; therefore, advancement decisions are best supported by multi-year summaries that represent performance across a target population of environments (Falconer and Mackay, 1996; Holland et al., 2003; Bernardo, 2020). Values reported as 0 should be interpreted as negligible detectable genetic variance under the fitted model (often reflecting sampling variability and/or large residual environmental variation), rather than evidence that the trait lacks a genetic basis (Holland et al., 2003).

The significant genotypic variation detected for pollen DAP indicates that flowering (phenological) timing in white maize is under strong genetic control and is a major driver of environmental adaptation. In contrast, the absence of significant genotype effects for plant height and ear height suggests that these traits were comparatively more buffered and/or more responsive to seasonal conditions than strictly determined by genotype in the present evaluation (Öztürk and Büyükgöz, 2021; Buckler et al., 2009; Huang et al., 2018; Du et al., 2021; Han et al., 2025).

Pronounced genotypic differences for grain yield indicate exploitable genetic diversity for whole-plant productivity under Black Sea conditions; however, the sizeable G×Y component shows that yield ranking is strongly season-dependent. Accordingly, selection based on a single year can be misleading and should rely on multi-year summaries complemented by stability assessment (Finlay and Wilkinson, 1963; Eberhart and Russell, 1966; Holland et al., 2003; Yan and Kang, 2003). In this context, the commercial check P2948W generally remained among the higher-yielding entries, while several testcrosses performed competitively, supporting their potential for advancement when yield stability and multi-trait performance are considered jointly (Yue et al., 2022).

Across years, ash, oil, protein, cellulose, and starch showed no significant genotype differences in the combined analysis, suggesting limited genetic differentiation for these compositional attributes under the current sampling and analytical intensity. By contrast, significant year effects indicate that seasonal conditions can markedly alter whole-plant composition, emphasizing the need to standardize harvest stage and keep sample handling/NIRS preparation strictly uniform to ensure valid cross-season comparisons (Di Marco et al., 2002; Khan et al., 2024; Reddersen et al., 2013; Jeong et al., 2024; Mut et al., 2022). Because the evaluated entries represent a relatively small, intensively selected elite subset, repeated selection may have narrowed the genetic base, which can reduce observable among-genotype contrasts for some traits (Hufford et al., 2012; Kadoumi et al., 2025).

The GT-biplot analysis provided an integrated overview of the relationships among agronomic, quality, and mineral traits, enabling a comprehensive assessment of genotype performance and trait associations. The close alignment of phenological traits with plant height and ear height, together with minerals such as Mn and Mg, suggests coordinated trait expression, consistent with previous multivariate studies in maize (Özata, 2020; Han et al., 2024). In addition, the ideal-genotype view facilitated the identification of genotypes with balanced multi-trait performance, underscoring the utility of biplot-based approaches for supporting breeding decision-making (Kendal, 2020; Hosseini et al., 2025; Temizgül et al., 2024).

The strong agreement among the GT-biplot, AMMI, and GGE results supports the robustness of multivariate tools for evaluating yield performance and stability. The AMMI approach effectively separated the additive main effects from the multiplicative component of the genotype × year interaction (with years treated as environments), whereas GGE biplots clearly distinguished high-yielding, stable genotypes and identified year-specific winners (Azrai et al., 2023; Saed-Moucheshi et al., 2024; Ghazy et al., 2025). The consistent identification of TTBYM2019-12, TTBYM2019-11, and TTBYM2019–14 as both high-yielding and stable across multiple years highlights their potential for broad adaptation and sustained cultivation (Habib et al., 2024; Kendal, 2019). Overall, these analyses revealed pronounced genotype-specific variation in agronomic performance, yield stability, and mineral accumulation among the evaluated white maize genotypes. Integrating multi-year field data with multivariate and stability analyses therefore provides a strong basis for selecting genotypes that combine high productivity with reliable performance under variable conditions, offering practical guidance for breeding programs targeting improved yield stability and balanced multi-trait performance.

## 5 Conclusions

Across five years (2020–2024), the evaluated elite white maize materials showed clear genetic variation for yield-related performance, but genotype × year interaction strongly affected trait expression, confirming the need for multi-year selection under Black Sea conditions. Heritability results supported this pattern, with yield showing the highest repeatability across years (H² = 0.623) and pollen DAP moderate repeatability (H² = 0.472), while compositional traits were largely shaped by seasonal effects and showed limited genetic separation in the combined analysis. Concordant results from GT-biplot, AMMI, and GGE analyses enabled reliable identification of high-performing and stable candidates, with TTBYM2019-11, TTBYM2019-12, and TTBYM2019–14 consistently emerging as promising genotypes. Overall, integrating multi-year field testing with stability and multi-trait analyses provides a robust basis for advancing productive, stable white maize cultivars for Türkiye’s Black Sea region and the national cultivar registration pipeline.

## Data Availability

The original contributions presented in the study are included in the article/**Supplementary Material**. Further inquiries can be directed to the corresponding authors.
