# Supplementary material for: Multiyear evaluation of agronomic traits, nutritional quality, macro and microelement profiles of white maize genotypes (Zea mays L.) under Black Sea conditions
Source: Front Plant Sci. 2026 Mar 18;17:1793293. doi: 10.3389/fpls.2026.1793293 (PMC13038609; doi:10.3389/fpls.2026.1793293)
Supplement: Supplementary file 1 [file DataSheet1.docx]

**Supplementary Figure 1.** Geographic distribution of the sampling sites of local white maize (*Zea mays* L.) populations collected across the Black Sea region of Türkiye. Blue circles indicate collection localities (latitude/longitude provided in Table S1). The inset map (bottom right) shows the location of Türkiye within Europe.


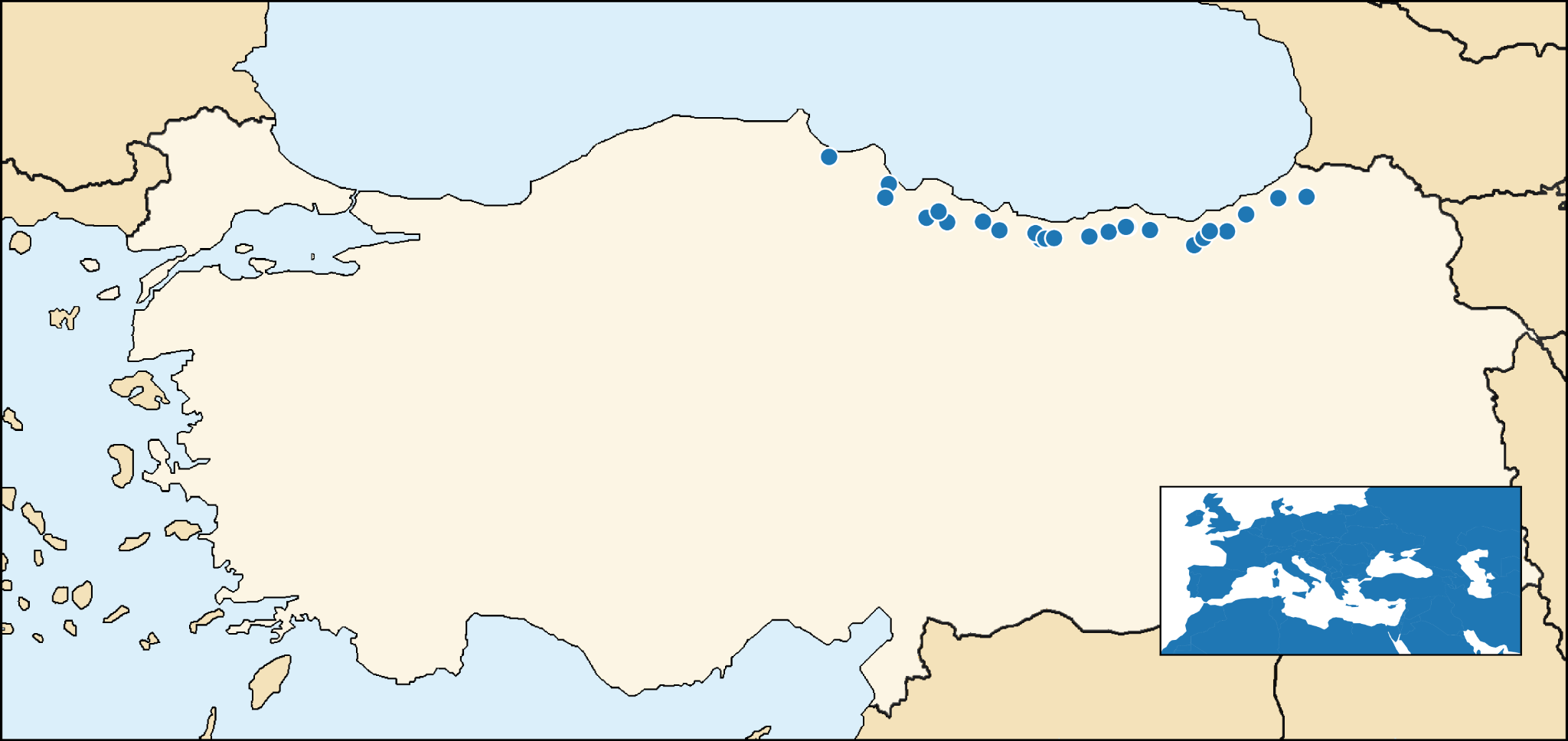


| City | District | Neighbourhood/bay | Latitude | Longitude |
| --- | --- | --- | --- | --- |
| Samsun | Bafra | Elifli | 41,506108 | 35,989816 |
|  | Atakum | Nebiyan | 41,359494 | 35,938181 |
|  | Tekkeköy | Büyüklü | 41,144091 | 36,502916 |
|  | Salı Pazarı | Alanyaykın | 41,094629 | 36,784565 |
|  | Çarşamba | Kuşhane | 41,211284 | 36,666966 |
| Sinop | Gerze | Abdaloğlu | 41,798235 | 35,170393 |
| Ordu | Ünye | Döşemedibi | 41,100344 | 37,276805 |
|  | Fatsa | Hamlık | 41,009286 | 37,49939 |
|  | Gülyalı | Kestane | 40,912437 | 38,062176 |
|  | Altınordu | Turnasuyu | 40,976349 | 37,995197 |
| Giresun | Piraziz | Nevsipiraziz | 40,919844 | 38,128507 |
|  | Bulancak | Uçarlı | 40,924021 | 38,248962 |
|  | Espiye | Hacıköy | 40,942102 | 38,731995 |
|  | Görele | Dedeli | 40,992006 | 38,994294 |
| Trabzon | Vakfıkebir | Körez | 41,044763 | 39,260007 |
|  | Sürmene | Ormanseven | 40,849581 | 40,16497 |
|  | Of | Kaban | 40,926808 | 40,291327 |
|  | Beşikdüzü | Beşikdağ | 41,044654 | 39,229815 |
|  | Akçaabat | Ağaçlı | 41,011769 | 39,559622 |
| Rize | Güneysu | Köseler | 40,998751 | 40,612232 |
|  | İyidere | Yapraklar | 40,999908 | 40,378311 |
|  | Pazar | Soğuksu | 41,180832 | 40,874064 |
| Artvin | Arhavi | Yukarıhacılar | 41,354316 | 41,313718 |
|  | Borçka | Arkaköy | 41,367378 | 41,699265 |

**Supplementary Table 1:** Geographic origin of the local white maize (*Zea mays* L.) populations collected from the Black Sea region of Türkiye. The table reports the sampling location for each population at the administrative level (province/city, district, and village/neighbourhood) together with the corresponding latitude and longitude coordinates. Coordinates are presented in decimal degrees (WGS84) to enable precise localization and reproducibility of the sampling sites.

| **Trait** | **Year** | **MS_G** | **MS_e** | **sigma_G** | **sigma_e** | **Reps (r)** | **H2_within_year** |
| --- | --- | --- | --- | --- | --- | --- | --- |
| Pollen DAP | 2020 | 16,43132 | 6,343407 | 5,043956 | 6,343407 | 2 | 0,613944 |
| Pollen DAP | 2021 | 0,936813 | 0,431319 | 0,252747 | 0,431319 | 2 | 0,539589 |
| Pollen DAP | 2022 | 6,936813 | 5,112637 | 0,912088 | 5,112637 | 2 | 0,26297 |
| Pollen DAP | 2023 | 2,343407 | 2,035714 | 0,153846 | 2,035714 | 2 | 0,131301 |
| Pollen DAP | 2024 | 24,28571 | 8,384615 | 7,950549 | 8,384615 | 2 | 0,654751 |
| Plant Height (cm) | 2020 | 376,1676 | 146,4973 | 114,8352 | 146,4973 | 2 | 0,610553 |
| Plant Height (cm) | 2021 | 288,1868 | 251,6484 | 18,26923 | 251,6484 | 2 | 0,126787 |
| Plant Height (cm) | 2022 | 530,2198 | 341,2088 | 94,5055 | 341,2088 | 2 | 0,356477 |
| Plant Height (cm) | 2023 | 111,8819 | 574,5192 | 0 | 574,5192 | 2 | 0 |
| Plant Height (cm) | 2024 | 356,6621 | 318,75 | 18,95604 | 318,75 | 2 | 0,106297 |
| Ear Height (cm) | 2020 | 127,2665 | 53,09066 | 37,08791 | 53,09066 | 2 | 0,582839 |
| Ear Height (cm) | 2021 | 151,1676 | 202,2665 | 0 | 202,2665 | 2 | 0 |
| Ear Height (cm) | 2022 | 147,5275 | 108,5165 | 19,5055 | 108,5165 | 2 | 0,264432 |
| Ear Height (cm) | 2023 | 122,8022 | 224,7253 | 0 | 224,7253 | 2 | 0 |
| Ear Height (cm) | 2024 | 307,1429 | 130,7692 | 88,18681 | 130,7692 | 2 | 0,57424 |
| Kernel /Cob Ratio (%) | 2020 | 0 | 0 | 0 | 0 | 2 | 0 |
| Kernel /Cob Ratio (%) | 2021 | 2,382005 | 0,543984 | 0,919011 | 0,543984 | 2 | 0,771628 |
| Kernel /Cob Ratio (%) | 2022 | 3,240994 | 1,785268 | 0,727863 | 1,785268 | 2 | 0,44916 |
| Kernel /Cob Ratio (%) | 2023 | 2,583632 | 1,092498 | 0,745567 | 1,092498 | 2 | 0,577147 |
| Kernel /Cob Ratio (%) | 2024 | 2,945949 | 1,345498 | 0,800225 | 1,345498 | 2 | 0,543272 |
| Moisture (%) | 2020 | 8,485852 | 8,186731 | 0,14956 | 8,186731 | 2 | 0,035249 |
| Moisture (%) | 2021 | 5,323736 | 5,277253 | 0,023242 | 5,277253 | 2 | 0,008731 |
| Moisture (%) | 2022 | 3,082198 | 1,199011 | 0,941593 | 1,199011 | 2 | 0,610988 |
| Moisture (%) | 2023 | 1,432665 | 2,075742 | 0 | 2,075742 | 2 | 0 |
| Moisture (%) | 2024 | 14,13201 | 4,009588 | 5,061209 | 4,009588 | 2 | 0,716276 |
| Yield (t/ha) | 2020 | 639,2507 | 59,62305 | 289,8138 | 59,62305 | 2 | 0,90673 |
| Yield (t/ha) | 2021 | 689,7438 | 63,81637 | 312,9637 | 63,81637 | 2 | 0,907478 |
| Yield (t/ha) | 2022 | 242,8869 | 206,1162 | 18,38531 | 206,1162 | 2 | 0,15139 |
| Yield (t/ha) | 2023 | 1059,433 | 38,20143 | 510,6159 | 38,20143 | 2 | 0,963942 |
| Yield (t/ha) | 2024 | 406,4068 | 384,8219 | 10,79243 | 384,8219 | 2 | 0,053111 |
| Ash | 2020 | 0,026112 | 0,106097 | 0 | 0,106097 | 2 | 0 |
| Ash | 2021 | 0,124868 | 0,105921 | 0,009473 | 0,105921 | 2 | 0,151735 |
| Ash | 2022 | 0,026072 | 0,100235 | 0 | 0,100235 | 2 | 0 |
| Ash | 2023 | 0,605523 | 0,233347 | 0,186088 | 0,233347 | 2 | 0,614636 |
| Ash | 2024 | 0,276581 | 0,401524 | 0 | 0,401524 | 2 | 0 |
| Fat | 2020 | 0,066208 | 0,075552 | 0 | 0,075552 | 2 | 0 |
| Fat | 2021 | 0,051084 | 0,129782 | 0 | 0,129782 | 2 | 0 |
| Fat | 2022 | 0,049392 | 0,108044 | 0 | 0,108044 | 2 | 0 |
| Fat | 2023 | 0,068578 | 0,111913 | 0 | 0,111913 | 2 | 0 |
| Fat | 2024 | 0,04524 | 0,119712 | 0 | 0,119712 | 2 | 0 |
| Protein | 2020 | 1,619686 | 2,055689 | 0 | 2,055689 | 2 | 0 |
| Protein | 2021 | 1,269107 | 1,715206 | 0 | 1,715206 | 2 | 0 |
| Protein | 2022 | 3,987116 | 2,573088 | 0,707014 | 2,573088 | 2 | 0,35465 |
| Protein | 2023 | 1,764814 | 1,280694 | 0,24206 | 1,280694 | 2 | 0,274318 |
| Protein | 2024 | 0,939126 | 0,688185 | 0,12547 | 0,688185 | 2 | 0,267206 |
| Cellulose | 2020 | 0,914454 | 0,635638 | 0,139408 | 0,635638 | 2 | 0,3049 |
| Cellulose | 2021 | 0,641685 | 1,109798 | 0 | 1,109798 | 2 | 0 |
| Cellulose | 2022 | 1,35351 | 0,774147 | 0,289681 | 0,774147 | 2 | 0,428045 |
| Cellulose | 2023 | 0,918707 | 1,262805 | 0 | 1,262805 | 2 | 0 |
| Cellulose | 2024 | 1,29871 | 1,242992 | 0,027859 | 1,242992 | 2 | 0,042903 |
| Starch | 2020 | 11,5112 | 3,641233 | 3,934982 | 3,641233 | 2 | 0,683679 |
| Starch | 2021 | 7,02097 | 3,534996 | 1,742987 | 3,534996 | 2 | 0,496509 |
| Starch | 2022 | 8,56442 | 6,676635 | 0,943893 | 6,676635 | 2 | 0,220422 |
| Starch | 2023 | 12,79398 | 4,028355 | 4,382812 | 4,028355 | 2 | 0,685137 |
| Starch | 2024 | 7,666436 | 7,947243 | 0 | 7,947243 | 2 | 0 |
| Fe (ppm) | 2020 | 18,87266 | 28,90487 | 0 | 28,90487 | 2 | 0 |
| Fe (ppm) | 2021 | 26,99641 | 19,77656 | 3,609924 | 19,77656 | 2 | 0,267437 |
| Fe (ppm) | 2022 | 24,20415 | 20,99851 | 1,602823 | 20,99851 | 2 | 0,132442 |
| Fe (ppm) | 2023 | 30,52987 | 27,58459 | 1,472643 | 27,58459 | 2 | 0,096472 |
| Fe (ppm) | 2024 | 14,04654 | 18,05314 | 0 | 18,05314 | 2 | 0 |
| Cu (ppm) | 2020 | 3,794882 | 0,652637 | 1,571122 | 0,652637 | 2 | 0,828022 |
| Cu (ppm) | 2021 | 2,193699 | 0,517161 | 0,838269 | 0,517161 | 2 | 0,764252 |
| Cu (ppm) | 2022 | 1,52735 | 0,377858 | 0,574746 | 0,377858 | 2 | 0,752605 |
| Cu (ppm) | 2023 | 2,328126 | 0,66708 | 0,830523 | 0,66708 | 2 | 0,713469 |
| Cu (ppm) | 2024 | 1,231717 | 0,364018 | 0,433849 | 0,364018 | 2 | 0,704463 |
| Mn (ppm) | 2020 | 1,802821 | 1,328945 | 0,236938 | 1,328945 | 2 | 0,262853 |
| Mn (ppm) | 2021 | 2,431076 | 1,683993 | 0,373542 | 1,683993 | 2 | 0,307305 |
| Mn (ppm) | 2022 | 1,457774 | 0,806714 | 0,32553 | 0,806714 | 2 | 0,446612 |
| Mn (ppm) | 2023 | 1,990622 | 1,841272 | 0,074675 | 1,841272 | 2 | 0,075027 |
| Mn (ppm) | 2024 | 1,398699 | 1,872216 | 0 | 1,872216 | 2 | 0 |
| Ca (ppm) | 2020 | 36217,08 | 54485,4 | 0 | 54485,4 | 2 | 0 |
| Ca (ppm) | 2021 | 316691,5 | 299608,3 | 8541,587 | 299608,3 | 2 | 0,053943 |
| Ca (ppm) | 2022 | 46310,2 | 83499,28 | 0 | 83499,28 | 2 | 0 |
| Ca (ppm) | 2023 | 259572,1 | 328364,1 | 0 | 328364,1 | 2 | 0 |
| Ca (ppm) | 2024 | 48776,84 | 110679,2 | 0 | 110679,2 | 2 | 0 |
| Mg (ppm) | 2020 | 49961,46 | 23005,26 | 13478,1 | 23005,26 | 2 | 0,53954 |
| Mg (ppm) | 2021 | 22341,6 | 16149,99 | 3095,805 | 16149,99 | 2 | 0,277134 |
| Mg (ppm) | 2022 | 21136,6 | 13836,18 | 3650,211 | 13836,18 | 2 | 0,345392 |
| Mg (ppm) | 2023 | 24880,81 | 21062,02 | 1909,396 | 21062,02 | 2 | 0,153483 |
| Mg (ppm) | 2024 | 21968,28 | 14342,34 | 3812,97 | 14342,34 | 2 | 0,347134 |
| K (ppm) | 2020 | 471558,4 | 176407,9 | 147575,3 | 176407,9 | 2 | 0,625904 |
| K (ppm) | 2021 | 252938,7 | 77107,76 | 87915,46 | 77107,76 | 2 | 0,695152 |
| K (ppm) | 2022 | 229190,8 | 147027,8 | 41081,5 | 147027,8 | 2 | 0,358492 |
| K (ppm) | 2023 | 270292 | 115997,5 | 77147,25 | 115997,5 | 2 | 0,570844 |
| K (ppm) | 2024 | 253693,2 | 136159,7 | 58766,71 | 136159,7 | 2 | 0,46329 |
| P (ppm) | 2020 | 254255 | 104625,3 | 74814,86 | 104625,3 | 2 | 0,588503 |
| P (ppm) | 2021 | 113441,9 | 50718,47 | 31361,69 | 50718,47 | 2 | 0,552912 |
| P (ppm) | 2022 | 93433,72 | 67035,5 | 13199,11 | 67035,5 | 2 | 0,282534 |
| P (ppm) | 2023 | 165982,5 | 53457,89 | 56262,28 | 53457,89 | 2 | 0,67793 |
| P (ppm) | 2024 | 73480,25 | 68650,28 | 2414,986 | 68650,28 | 2 | 0,065732 |
| Zn (ppm) | 2020 | 19,4544 | 16,25476 | 1,599819 | 16,25476 | 2 | 0,164469 |
| Zn (ppm) | 2021 | 10,82202 | 7,222523 | 1,799749 | 7,222523 | 2 | 0,332609 |
| Zn (ppm) | 2022 | 6,920252 | 6,200708 | 0,359772 | 6,200708 | 2 | 0,103977 |
| Zn (ppm) | 2023 | 13,66228 | 10,90276 | 1,379762 | 10,90276 | 2 | 0,201981 |
| Zn (ppm) | 2024 | 8,117648 | 5,660174 | 1,228737 | 5,660174 | 2 | 0,302732 |

**Supplementary Table 2:** Year-specific variance components and within-year broad-sense heritability estimates for agronomic, grain quality, and mineral composition traits of white maize (*Zea mays* L.) evaluated over five years (2020–2024). For each trait and year, the table reports the genotype mean square (MS_G) and error mean square (MS_e), the corresponding estimated variance components for genotype (σ_G) and residual/error (σ_e), the number of replications (r), and the within-year broad-sense heritability (H²_within-year). H²_within-year was calculated as $H^{2}=\sigma_{G}/(\sigma_{G}+\sigma_{e}/r)$. Traits are presented with their original measurement units (e.g., cm, %, t ha⁻¹, ppm). Values of σ_G or H² equal to zero indicate negligible genotypic variance relative to residual variance in that year.
